# THE IMPACT OF CHILDHOOD ADVERSITY ON CAREGIVERS' HEALTH

**DOI:** 10.1093/geroni/igac059.664

**Published:** 2022-12-20

**Authors:** Elizabeth Avent, Jeanine Yonashiro-Cho, Zach Gassoumis

**Affiliations:** University of Southern California, Los Angeles, California, United States; Keck School of Medicine of USC, Alhambra, California, United States; Keck School of Medicine, USC, Los Angeles, California, United States

## Abstract

Caregivers tend to report fair or poor health compared to non-caregivers. Adverse childhood experiences (ACEs), which are traumatic events experienced before age 18, have been associated with several health conditions and overall poor health in adulthood. The additive effect of early-life stressors and caregiving stressors may have a compounded impact on the health of caregivers, contributing additional stress and burden to their caregiving situation. Data from the 2019 and 2020 Behavioral Risk Factor Surveillance System (BRFSS) were used to analyze self-rated health (SRH) in the context of caregiving and ACEs, based on responses from Florida, Georgia, Tennessee, Virginia, and Utah (N= 41,334). Of the 8,368 caregivers, nearly 23% reported 4 or more ACEs, compared with 13% of non-caregivers. Nested regression models showed that caregivers have lower SRH (b=-0.04, p=0.0002); however, after including the combination of ACEs and being a caregiver, there was no significant impact of being a caregiver alone on SRH. The effect of ACEs alone on SRH persisted, with the strongest effect for individuals with 4 or more ACEs (b=-0.41, p< 0001). The finding that caregivers’ childhood adversity accounts for SRH moreso than their caregiver status highlights the importance of a lifespan approach when considering caregiver health and, potentially, burden. This has implications for practice, suggesting that screening caregivers on their history of ACEs may be a valuable tool to identify caregivers at higher risk for poor health outcomes who may benefit from additional resources to support their health and well-being.

